# Glucose metabolic flux distribution of *Lactobacillus amylophilus* during lactic acid production using kitchen waste saccharified solution

**DOI:** 10.1111/1751-7915.12046

**Published:** 2013-03-14

**Authors:** Jianguo Liu, Qunhui Wang, Hui Zou, Yingying Liu, Juan Wang, Kemin Gan, Juan Xiang

**Affiliations:** 1 School of Civil and Environmental Engineering, University of Science and Technology Beijing30 Xueyuan Road, Haidian District, Beijing, 100083, China; 2 Key Laboratory of Educational Ministry for High Efficient Mining and Safety in Metal Mine, University of Science and Technology BeijingBeijing, 100083, China; 3 Biological laboratory, National Institute of Metrology P.C.ChinaBeijing, 100013, China

## Abstract

The ^13^C isotope tracer method was used to investigate the glucose metabolic flux distribution and regulation in *Lactobacillus amylophilus* to improve lactic acid production using kitchen waste saccharified solution (KWSS)*.* The results demonstrate that *L. amylophilus* is a homofermentative bacterium. In synthetic medium, 60.6% of the glucose entered the Embden–Meyerhof–Parnas (EMP) to produce lactic acid, whereas 36.4% of the glucose entered the pentose phosphate metabolic pathway (HMP). After solid–liquid separation of the KWSS, the addition of Fe^3+^ during fermentation enhanced the NADPH production efficiency and increased the NADH content. The flux to the EMP was also effectively increased. Compared with the control (60.6% flux to EMP without Fe^3+^ addition), the flux to the EMP with the addition of Fe^3+^ (74.3%) increased by 23.8%. In the subsequent pyruvate metabolism, Fe^3+^ also increased lactate dehydrogenase activity, and inhibited alcohol dehydrogenase, pyruvate dehydrogenase and pyruvate carboxylase, thereby increasing the lactic acid production to 9.03 g l^−1^, an increase of 8% compared with the control. All other organic acid by-products were lower than in the control. However, the addition of Zn^2+^ showed an opposite effect, decreasing the lactic acid production. In conclusion it is feasible and effective means using GC-MS, isotope experiment and MATLAB software to integrate research the metabolic flux distribution of lactic acid bacteria, and the results provide the theoretical foundation for similar metabolic flux distribution.

## Introduction

The composition of kitchen waste is very complex and it mainly consists of organic matter such as starch, dietary fibre, animal fats and so on. Kitchen waste has high moisture content, as well as a high fat, high salt and high starch content. Kitchen waste is mainly disposed through incineration and composting. Considering kitchen waste is not readily combustible, other fuels need to be mixed in. However, kitchen waste hinder the increase in incinerator temperature and the presence of chlorinated compounds corrodes the fireplace and produces dioxins. In addition, the long-term application of high-salt-content fertilizers made from kitchen waste causes adverse effects on the soil and on plant growth, which restricts the development of kitchen waste composting. Lactic acid is an organic acid that is popularly employed in many fields and is in huge demand. Polylactic acid plastics synthesized from lactic acid are biodegradable, environmentally benign, and have broad applications in the market (Yun *et al*., 2003). Preparing lactic acid from kitchen waste could solve the environmental problems caused by the waste itself, and the product can be utilized as an alternative to non-renewable fossil resources to reduce the ‘white pollution’ caused by the combustion of non-degradable plastics (Wang *et al*., 2005).

Current studies on lactic acid production from food waste fermentation focus on the optimization of the fermentation process and screening of the target flora. Kim investigated the simultaneous saccharification and fermentation of food waste to produce lactic acid using glucoamylase, α-amylase, protease and *Lactobacillus delbrueckii* (Kim *et al*., 2003). Wang also conducted thorough studies on the bioconversion of kitchen waste into lactic acid through the open fermentation process of simultaneous saccharification and fermentation (Wang *et al*., 2003; Wang and Wang, 2005). Jiang *et al*. isolated a strain of heat-resistant lactic acid bacteria, TY50, from the anaerobic fermentation of kitchen waste, which produced up to 36.29 g l^−1^ of lactic acid through kitchen waste fermentation (Jiang *et al*., 2008). However, metabolic flux distribution analysis in relation to lactic acid production through food waste fermentation has not been reported.

At present, analysing the metabolic flux distribution is widely used for amino acid-producing bacteria to evaluate the changes in the metabolic phenotype under different conditions, thereby fermentation process would be optimized, such as the optimization for glutamic acid and lysine production (Vallino and Stephanopoulos, 1992; Takac *et al*., 2005; Gheshlaghi *et al*., 2007). Novak and Loubiere adopted ^14^C glucose to study the glucose metabolic pathway and its flow changes in *Lactococcuslactis* (NCDO 2118) in synthetic medium containing only six essential amino acids and glucose as the sole carbon source (Novak and Loubiere, 2000). Furch studied the central metabolic fluxes of *Bacillus megaterium* strains in continuous culture using ^13^C-labelled substrates (Furch *et al*., 2007). Scandellari studied lipid biosynthesis pathways in ectomycorrhizal fungi through the position-specific ^13^C labelling of glucose (Scandellari *et al*., 2009). To obtain the highest lactic acid yield, researchers used additional inhibitory substances such as mannitol, to inhibit the enzyme activity involved in ethanol metabolism, thereby promoting the carbon flux for lactic acid (Bhatt and Srivastava, 2008). *Lactobacillus amylophilus* is a strain of lactic acid bacteria capable of hydrolysing starch, the amylase produced by this strain has both amylase and amylopectase activity, could convert complex starch substrates into lactic acid (Zhao *et al*., 2009). However, there existed no research on lactic acid production from kitchen waste by *L. amylophilus*, and the metabolic flux during this process was also worth investigating. Trace elements maintain the functions of many enzymes and transcription factors, and they also play important roles in cell growth, development and differentiation. Nitrogen is the basic element in the formation of amino acids, purines, pyrimidines and so on. In the current study, the trace elements Fe^3+^ and Zn^2+^, and a nitrogen source (sodium nitrate) were added to possibly change the metabolic flux distribution and determine the flow of glucose as a carbon source in lactic acid formation (Feng *et al*., 2009).

The current study aims to determine the metabolic flux of *L. amylophilus* in kitchen waste saccharified solution (KWSS) to produce lactic acid using ^13^C-MFA. Trace elements were added to the system to optimize the metabolic flux distribution of the Embden–Meyerhof–Parnas (EMP; glycolysis) pathway during fermentation, thus providing a theoretical basis for the efficient use of food waste.

## Results and discussion

### Metabolic type analysis of *L. amylophilus* on MRS medium

**Figure 1:**
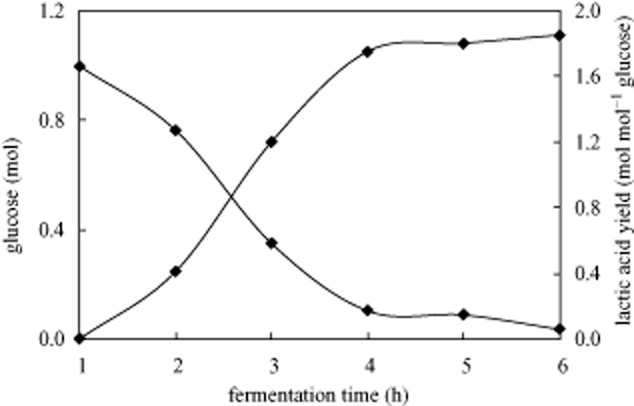
Lactic acid yield of *Lactobacillus amylophilus* on MRS medium (37°C).

Generally, three metabolic pathways are observed in lactic acid bacteria, homofermentation, heterofermentation and the bifidum pathway. In homofermentation, 1 mol of glucose generates 2 mol of lactate and 2 mol of ATP (see Eq. 1). Homofermentation is generally considered when the glucose to lactic acid conversion rate exceeds 80% of the theoretical yield. *Lactobacillus amylophilus* was subjected to a metabolic experiment on labelled ^13^C MRS medium (glucose as the carbon source) to determine the pathway it used in fermentation and to establish a foundation for its flux calculation. The results are shown in Fig. 1. Anaerobic digestion of the ^13^C-labelled MRS medium resulted in the rapid growth of *L. amylophilus*. Lactate production gradually increased and reached a steady conversion rate after 12 h. Although very small amounts of formic acid, acetic acid and tartaric acid were formed as fermentation products, the conversion rate of glucose to lactic acid was still much higher than the other pathways, reaching (1.85–1.95) mol mol^−1^ of glucose (Fig. 1). This accounts for 92.5% to 97.5% of the theoretical conversion rate (> 80%), which demonstrates that *L. amylophilus* undergoes homofermentation. This result also indicates that *L. amylophilus* has the potential to metabolize complex substrates. Consequently, *L. amylophilus* was inoculated into KWSS and the metabolic flux distribution was investigated to optimize the metabolic flux and improve the lactic acid conversion rate.


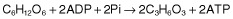
(1)

### Metabolic flux distribution and optimization of glucose metabolism in KWSS medium

Considering the amylase produced by *L. amylophilus* has both amylase and amylopectase activity, the starch in food waste can be used to generate lactic acid. The ^13^C-labelled fermentation was carried out according to the method described in the second paragraph of Isotope experiment. Liquid chromatography analysis indicated that the major organic acids in the fermentation broth (extracellular flow) were mainly lactic acid, formic acid, tartaric acid and acetic acid. The most abundant product of the fermentation was lactic acid at 8.34 g l^−1^, which accounted for 90.5% of the total organic acids. The formic acid, acetic acid and tartaric acid content were relatively much lower; all were less than 0.5 g l^−1^ and accounted for 4.56%, 3.04% and 1.95% of the total organic acids respectively. The content of the other organic acids were even lower, and negligible. Meanwhile, the concentration of amino acids on the synthetic medium was analysed. The 12 kinds of amino acids obtained were Thr, Ser, Pro, Gly, Ala, Val, Ile, Leu, Tyr, Phe, Lys and His, and their concentrations were (mmol l^−1^): 0.27, 0.48, 0.12, 0.25, 0.28, 0.38, 0.51, 1.18, 0.24, 1.24, 1.08 and 1.21 respectively.

**Figure 2:**
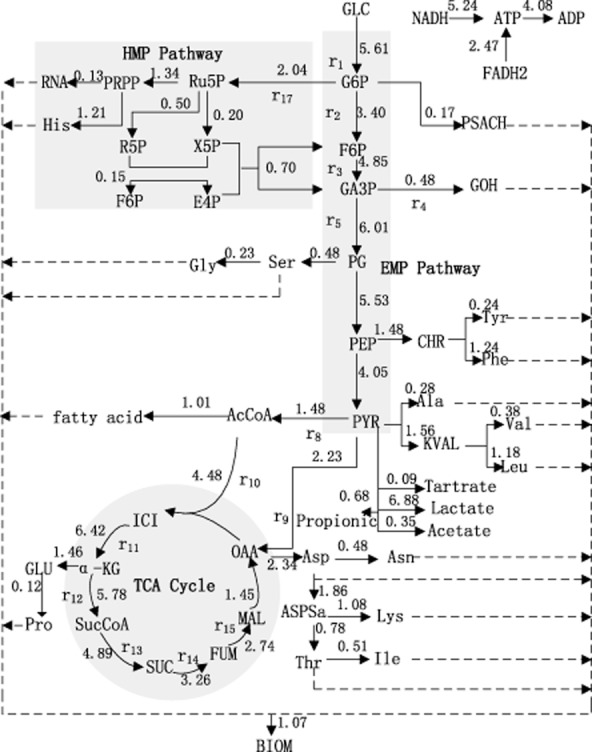
Metabolic flux distribution of *Lactobacillus amylophilus* in ^13^C-labelled KWSS.

The intracellular metabolic flux of *L. amylophilus* was obtained using the matrix in MATLAB based on the extracellular flow and amino acid data. As shown in Fig. 2, glucose was biodegraded by *L. amylophilus* through the pentose phosphate pathway (HMP) and the glycolytic pathway (EMP). In the KWSS, 36.7% of the metabolic flux entered the HMP pathway, whereas 60.6% entered the EMP pathway. The conversion rate of glucose to lactic acid through the EMP pathway was 86.6% (1.732 mol mol^−1^). The conversion rate with KWSS was slightly lower than that in MRS medium (1.85 mol mol^−1^ to 1.95 mol mol^−1^), but it was still greater than 80%; thus, the bacteria underwent homofermentation. These results indicate the feasibility of using *L. amylophilus* to produce lactic acid from KWSS.

On account of Fe^3+^ is related to an inefficient glucose utilization pathway, whereas Zn^2+^ is a common factor that naturally exists in cellular proteins, nucleic acids, carbohydrates and lipids, and its presence directly affects cellular reaction. The change in metabolic flux distribution after the control experiment (without any external material), the addition of trace elements and the nitrogen source is shown in Table 1. To reduce the complexity of metabolic networks, only the main metabolic flux balance is shown.

**Table 1:** Metabolic flux distributions of glucose after addition of Fe^3+^, Zn^2+^ and NaNO_3_ in KWSS (%)

	Glucose						
	
	Pyruvate (EMP)						
		
	Tartaric acid	Propionic acid	Lactic acid	Acetic acid	Ethanol and other	Total	Pentose (HMP)
Contrast	1.79	2.45	52.48	3.05	0.82	60.60	36.70
Zn^2+^	2.35	3.53	44.42	2.72	1.08	54.10	45.00
Fe^3+^	0.82	1.54	70.36	0.76	0.75	74.30	25.00
NaNO_3_	0.66	3.65	63.37	1.40	1.02	70.10	28.90

As shown in Table 1, the addition of Fe^3+^ to KWSS leads to the highest possible lactic acid conversion rate 70.36% (94.7% proportion of pyruvate way). Compared with the systems supplied with Zn^2+^ and NaNO_3_ and with the control, the conversion rate in the system with Fe^3+^ was higher by 17.8%, 5.64% and 9.32% respectively. The addition of NaNO_3_ also increased the conversion rate, but the change was not as obvious as with Fe^3+^. After the addition of Zn^2+^, the metabolic flux into HMP pathway increased by 38.9%, whereas the flux into the EMP pathway decreased, thus decreasing the lactic acid flux. Comparison of the organic acid conversion rates in the fermentation broth under four different conditions revealed that the addition of Fe^3+^ effectively inhibits the generation of by-products. Acetic and propionic acid production reached their minimum and the flux to the HMP pathway was minimal. The lactic acid production is 9.03 g l^−1^, and conversion rate compared with the contrast increase by 8.3% to 90.3%. In contrast, the addition of Zn^2+^ inhibited lactic acid generation and increased the production of the undesired products (propionic and tartaric acids). The addition of the trace element Zn^2+^ did not produce the expected increase in lactic acid flux. The possible reasons for the changes in the key enzymes and coenzymes of *L. amylophilus* caused by the addition of Fe^3+^ and Zn^2+^ are discussed in subsequent sections.

### Effects of Fe^3+^ on key enzymes and coenzymes

In lactic acid bacteria, the key coenzyme reduced form of nicotinamide-adenine dinucleotid (NADH) and nicotinamide adenine dinucleotide phosphate (NADPH), greatly affects the decomposition of carbon source and the direction of metabolic synthesis. In *L. amylophilus*, NADPH is needed to provide the reducing power for biosynthesis and promote pyruvate production to synthesize cellular component material (amino acids, nucleotides and lipids among others) using glucose and intermediates, thereby providing raw materials for further lactic acid production. Two pathways in *L. amylophilus* produce NADPH, glucose-6-phosphate acid dehydrogenation in the HMP pathway and isocitrate dehydrogenation in TCA cycle. The amounts of coenzyme NADH generated and consumed are shown in Table 2. The total amount of NADPH after the addition of Fe^3+^ was 26.6% higher than the control. On the other hand, the HMP/TCA ratio was 10.8% lower than the control even though the flux of pyruvate into TCA cycle (r_8_) changed little compared with the control. This demonstrates that the addition of Fe^3+^ activates the TCA and produces more NADPH. This further increase in NADPH production is probably because Fe^3+^ enhances the ability of the cells to adjust the NADPH production automatically to satisfy its needs for biosynthesis. The results indicate that Fe^3+^ promotes the flux to pyruvate synthesis (EMP pathway) and NADPH yield, consequently optimizing the metabolic flux distribution.

**Table 2:** Effect of Fe^3+^ on flux distribution of NADPH and NADH

	Flux distribution of NADPH generation			Generation and consumption of NADH			
		
	HMP/TCA (%)^a^	HMP/(HMP + TCA) (%)^b^	Total NADPH flux^c^	From EMP (%)^d^	From TCA cycle (%)^e^	Forming glycerol (%)^f^	Total NADH flux^g^
Control	28.6	22.3	3.12	52.1	45.3	5.48	6.32
Fe^3+^	17.8	15.1	3.95	73.2	24.2	4.13	7.83
Variation	-10.8		+26.6%	+40.5			+23.9%

a HMP/TCA = (r_17_/3)/(r_11_/6) × 100%, flux ratio of NADPH generated through HMP (r_17_) and isocitrate dehydrogenation in TCA cycle (r_11_).

b HMP/(HMP + TCA) = (r_17_/3)/(r_11_/6 + r_17_/3) × 100%, proportion of NADPH generated through HMP (r_17_) in the total flux of NADPH (through the PPP and the TCA cycle).

c NADPH flux generated through reactions r_17_ and r_11_.

d Proportion of NADH flux generated through EMP (r_5_) in the total NADH flux.

r Proportion of NADH flux generated by the TCA cycle (r_8_ + r_12_ + r_13_) in the total NADH flux.

f Proportion of NADH flux to form glycerol through PDC pathway (r_4_) in the total NADH flux.

g Standardized by glucose consumption.

As shown in Fig. 2, after the addition of Fe^3+^ to the KWSS, the flux from node GA3P to glycerol (r_4_) decreased. The flux to PG (r_5_), the NADH-generating reaction in the EMP pathway, increased. Consequently, the amount of NADH generated from EMP increased, which accounted for 73.2% of the total NADH, 40.5% higher than that of the control. The flux to pyruvate in the TCA cycle (r_8_) decreased slightly compared with the control after the addition of Fe^3+^, which indicates that NADH consumption in the TCA cycle decreased. Given that lactic acid production from pyruvate needs to consume a large amount of NADH and the addition of Fe^3+^ increases the NADH generation and decreases NADH consumption in the TCA cycle, the increase in NADH promotes lactic acid production. This confirms that Fe^3+^ optimizes the sugar metabolic pathways by increasing the amount of available coenzyme, and that intracellular NADH plays an important role in metabolism of *L. amylophilus* to produce lactic acid from pyruvate.

**Figure 3:**
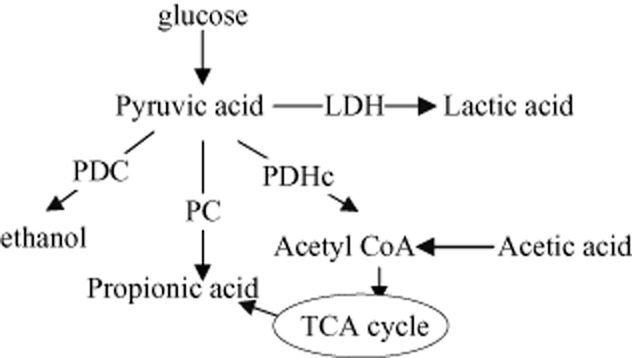
Key enzymes related to lactic acid synthesis pathway.

Figure 3 shows the key enzymes in lactic acid biosynthesis. The change in the activity of these enzymes directly affects lactic acid formation. Promoting LDH activity and inhibiting the activity of PDC, PC and PDHc are effective strategies for optimizing the metabolic pathway to increase lactic production during fermentation.

**Figure 4:**
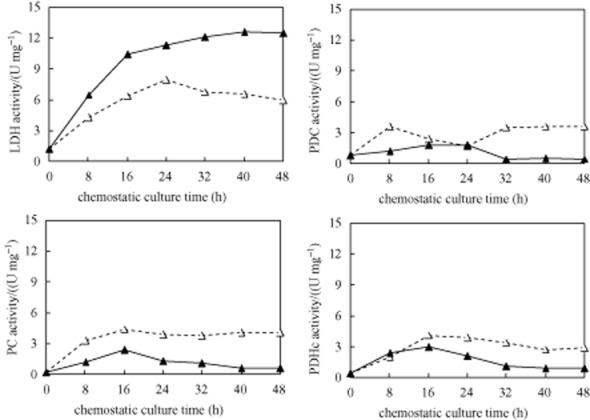
Effect of Fe^3+^ on the activity of LDH, PDC, PC and PDHc. Dashed lines represent the control.

Figure 4 shows the changes in the activity of the four key enzymes relative to fermentation time. The LDH activity in the KWSS system supplied with Fe^3+^ was higher than that of the control (Fig. 4A). After 48 h of fermentation, the highest enzyme activity, 12.08 U mg^−1^, was obtained, which is 53.11% higher than the highest activity in the control. Moreover, the addition of Fe^3+^ effectively inhibited the activity of PDC, PC and PDHc. The activity of the three enzymes were all lower than in the control, which indicates that Fe^3+^ is an effective promoting factor of *L. amylophilus* lactic acid fermentation.

### Effects of Zn^2+^ on key enzymes and coenzyme

Although the percentage of NADPH flux in the HMP pathway increased after the addition of Zn^2+^, NADPH production was almost unchanged and the total amount of NADPH was even lower than the control (Table 3). Thus, the addition of Zn^2+^ did not increase the NADPH yield and did not promote pyruvate generation. When Zn^2+^ was added to the KWSS system, the flux to r_17_ (HMP pathway) increased. Therefore, although the flux to glycerol from GA3P node (r_4_) decreased, the flux to PG (r_5_) but declined. Despite the increase in the NADH flux through the EMP pathway, its actual content did not increase. NADH formation through TCA cycle significantly decreased. This change was because the flux to TCA cycle decreased significantly and the addition of Zn^2+^ inhibited the activity of this cycle, thereby reducing NADH generation. The inhibition was also the reason why Zn^2+^ inhibited lactic acid production in *L. amylophilus*.

**Table 3:** Effect of Zn^2+^ on flux distribution of NADPH and NADH

	Flux distribution of NADPH generation			Generation and consumption of NADH			
		
	HMP/TCA (%)^a^	HMP/(HMP + TCA) (%)^b^	Total NADPH flux^c^	From EMP (%)^d^	From TCA cycle (%)^e^	Forming glycerol (%)^f^	Total NADH flux^g^
Control	28.6	22.3	3.12	52.1	45.3	5.48	6.32
Zn^2+^	31.7	24.6	2.68	60.5	37.1	5.78	5.41

The symbols in Table 3 are the same as in Table 2.

**Figure 5:**
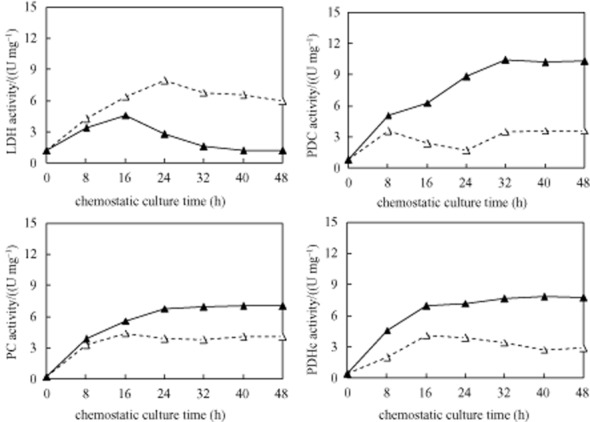
Effect of Zn^2+^ on the activity of LDH, PDC, PC and PDHc. Dashed lines represent the control.

In contrast to the system supplied with Fe^3+^, the LDH activity in the system supplied with Zn^2+^ was lower than the control, and the activity of PDC, PC and PDHc were higher (Fig. 5), which decreases the lactic acid conversion rate and increases the conversion rate of heteroacids and alcohols (Table 1). Therefore, the addition of Zn^2+^ to lactic acid fermentation system is unsuitable for *L. amylophilus*. However, this also suggests that Zn^2+^ significantly affects microbial activity and it could be considered as an inhibitor used in other similarly fermentation systems.

## Conclusions

The ^13^C-labelled experiments on the metabolic flux distribution of *L. amylophilus* indicate that it is a homofermentative bacterium and has the capacity to metabolize complex substrates. When *L. amylophilus* was inoculated into KWSS, the conversion rate of glucose to lactic acid was still greater than 80%, thus, the bacterium performs homofermentation.

The metabolic flux optimization experiments show that the addition of Fe^3+^ promotes the flux to pyruvate, thereby significantly increasing lactic acid production. The highest production, 9.03 g l^−1^, was achieved with the addition of Fe^3+^. In contrast, Zn^2+^ inhibits the flux to EMP pathway, significantly decreasing lactic acid production. The addition of NaNO_3_ promotes lactic acid production and increases the flux to EMP pathway, but this change is small and it does not significantly improve the metabolic flux.

Fe^3+^ promotes lactic acid fermentation in *L. amylophilus*. It increases the production of NADPH and NADH and promotes the flux to the EMP pathway and pyruvate generation. As a result, LDH activity increases, whereas the activity of PDC, PC and PDHc are inhibited, thereby increasing lactic acid production and inhibiting the generation of other organic acids. In contrast, Zn^2+^ reduces the production of NADPH and NADH and inhibits LDH activity, thereby decreasing lactic acid production.

## Experimental procedures

### Materials

The lactic acid bacteria *L. amylophilus* GV6 was purchased from the China General Microbiological Culture Collection Center.

Glucoamylase (10^5^ U g^−1^) was obtained from the Beijing Aoboxing Bio-Tech, whereas industrial-grade cellulose (270 U g^−1^) was bought from the Beijing Donghua Qiangsheng Bio-Tech.

The kitchen waste was obtained from the students' canteen of the University of Science and Technology, Beijing. After removing the non-biodegradable substances such as bone and plastic bags, the kitchen waste mainly consisted of rice, vegetables, meat, eggs, tofu, noodles and so on. The average starch content of the kitchen waste was 46.12%, and the protein content was 15.56%. The kitchen waste was stored and refrigerated at −20°C.

### Isotope experiment

The isotope experiment was performed on de Man–Rogosa–Sharpe (MRS) medium (0.8 g of U-^13^C glucose, 9.2 g of natural glucose, 2 g of Na_2_HPO_4_·2H_2_O, 0.1 g of MgSO_4_·7H_2_O, 0.04 g of MnSO_4_·H_2_O, 2.0 g of ammonium citrate and 1.0 ml of Tween-80, and sufficient distilled water to a final volume of 1000 ml). *Lactobacillus amylophilus* was inoculated into MRS medium at a final concentration of 2 g l^−1^. Anaerobic fermentation was carried out by filling the system with nitrogen gas.

Kitchen waste saccharification and fermentation was performed as follows: 1.5 g of glucoamylase and 12 g of cellulose enzyme were added to 100 g of food waste samples. The pH was adjusted to 5.0 and hydrolysis was performed at 50°C for 60 min. The glucose concentration reached 28.46 g l^−1^ after glycosylation. A saccharified solution was obtained after filtration. The saccharified solution was then diluted until its natural glucose (unlabelled) content was 9.2 g l^−1^. Then, 0.8 g l^−1^ labelled glucose was added to the diluted saccharified solution to a final ^13^C glucose ratio of 8.0%. The solution obtained was designated as kitchen waste saccharified solution (KWSS). *Lactobacillus amylophilus* was inoculated into the KWSS at 2 g l^−1^. Anaerobic fermentation was conducted by filling the system with nitrogen gas.

#### Principle of ^13^C labelling experiment

^13^C-MFA is a method to quantify the intracellular flux by measuring the extracellular flux and intracellular labelling information (i.e. intracellular fluxes = extracellular fluxes + labelling data). In ^13^C labelling experiments, ^13^C-labelled substrates (usually glucose) are introduced to the biological reaction system, and ^13^C atoms are distributed in extracellular and intracellular metabolites as well as biomass components via chemical rearrangement, rupture of chemical bonds and formation of new bonds in biological chemical reactions. Furthermore, along with the consumption of glucose, the isotopic abundance of labelled carbon atoms in the metabolites increases continuously. Once their abundance can be detected by nuclear magnetic resonance (NMR) or mass spectrometry (MS), samples are taken and analysed (Isermann and Wiechert, 2003).

Testing platform of ^13^C labelling experiments testing platform: the two main testing platforms for ^13^C labelling experiments are NMR test and MS test. NMR method started earlier and has a better test resolution, while the analysis of its spectrum is relatively difficult. MS test has a very high resolution and can obtain information of many trace labelled intermediate metabolites. Moreover, the spectrum analysis of MS is easier than that of NMR. Therefore, in this study, MS testing platform was chosen as the analysis and calculation method.

Metabolic flux optimization was performed using the following components: Fe^3+^ (0.204 mg l^−1^), Zn^2+^ (1.748 mg l^−1^) and NaNO_3_ (0.75 mg l^−1^) (Raynaud and Muriel, 2005). These were added to the KWSS containing ^13^C-glucose, and the changes in metabolic flux were investigated.

The metabolic network of lactic acid bacteria has been researched thoroughly so far, and themetabolic network distribution of homolactic acid bacteria has also been obtained (Bustos *et al*., 2005). *Lactobacillus amylophilus*, one kind of homolactics, was used in this study, and its metabolic network is expected to have some similarities with that obtained in former research. Therefore, in this study, the thorough metabolic network of *L. amylophilus* was obtained on this basis by analysing the extracellular and intracellular substances. The metabolic flux of this network is calculated according to the principle of mass conservation. The material balance of intermediate metabolites was obtained using quasi-steady assumption (Kauffman *et al*., 2003): assuming that all the intermediate metabolites are in quasi-steady state (i.e. the change rates of their concentration are zero), *n* different intermediate metabolites can get *n* constraint conditions. If the number of the rates selected is *J*, the freedom degree of problems to be solved (*F*) was calculated as (*J* − *n*). By this means, the overall flux distribution could be determined by measuring *F* uncorrelated rates.

The accumulation rate of metabolite *i* (*x_i_*) in the metabolic network was quantified using the following equation:


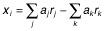
(2)

where *r_j_* is the production flux of metabolite *i* in reaction *j*; *r_k_* is the consumption flux of metabolite *i* in reaction *k*; *a_j_* and *a_k_* are the stoichiometric coefficients; *x_i_*, *r_j_* and *r_k_* are the specific rates.

Based on the metabolic network model already known, metabolic reaction equations of corresponding number of reaction steps and equations with an equal number of unknown data can be designed. Under the known experimental conditions, the parameters that can be detected are the concentrations of glucose, organic acids, amino acids and biomass. The release rate of carbon dioxide can be obtained according to carbon balance. By using MATLAB software program, the metabolic reaction equations can be solved, and thus the network and flux distribution of glucose and lactic acid metabolism of lactic acid bacteria during kitchen waste fermentation can be constructed.

### Analytical methods

#### Sample pre-treatment

The fermentation broth was centrifuged at 4000 r.p.m. for 15 min. The supernatant liquid was collected and diluted tenfold, and filtered with a 0.45 μm mixed-fibre resin membrane. After ultrasonic degassing for 20 min, the sugar, amino acids and organic acids were measured.

#### Sugar analysis

Twenty microlitres of the sample after pre-treatment was injected into an LC-20A liquid chromatograph [Rezex RCM-Monosaccharide of Ca^2+^ (8%), 300 mm × 7.8 mm] with a refractive index detector. The mobile phase was a mixture of acetonitrile and ultrapure water at a ratio of 75:25 (v/v; ultrasonically degassed for 30 min), and a flow rate of 0.6 ml min^−1^. The operation temperature of the column was maintained at 80°C.

#### Amino acid analysis

One microlitre of the sample after pre-treatment was injected into the QP2010 GC-MS instrument. The GC column used was HP5 MS (30 m × 0.25 mm × 0.25 μm). The GC conditions were as follows: carrier gas (helium) at a flow rate of 1.0 ml min^−1^ and split ratio of 50:1; EI power with an ionization voltage of 70 eV; scan range of (10–550) amu; temperatures of the injector, transfer line, ion source and quadrupole were 280°C, 280°C, 230°C and 150°C respectively. The temperature of the column was maintained at 80°C for 2 min, increased to 280°C at a rate of 10°C min^−1^ and sustained for 40 min. The amino acid components and yields in the fermentation products were then analysed (Maczek *et al*., 2006; Furch *et al*., 2007).

#### Ethanol analysis

An SBA-40C biosensor was used to measure the ethanol content of the fermentation liquid.

#### Organic acid analysis

A liquid chromatography LC-20A with Inertsil ODS-SP column (5 μm × 250 mm × 6.0 mm) was used under the following conditions. The mobile phase was ultrapure water (pH was adjusted to 2.5 with phosphoric acid) at a flow rate of 0.8 ml min^−1^; the detector wavelength was UV210 nm; the injection volume was 20 μl; and the column temperature was 30°C (Liu *et al*., 2012).

#### Crude enzyme preparation

The fermentation broth was centrifuged at 8500 r.p.m. for 15 min, and the cells were collected and centrifugally washed three times with 0.1 mol l^−1^ potassium phosphate buffer (pH 6.5). The cells were then resuspended in buffer and oscillated with glass beads at 4°C for 5 min to homogenize the cells. Subsequently, the cells were lysed through ultrasonication (KS-250) for 5 min in an ice bath. The work intensity of the instrument was at 30% for 1 s at an interval of 0.5 s. Finally, the liquid was centrifuged at 8000 r.p.m. for 15 min at 4°C. The resulting supernatant liquid was the crude enzyme solution.

#### Activity assay of the key enzymes (Peng *et al*., 2001)

Four enzymes that are closely related to lactic acid synthesis in glucose metabolism were selected, namely, lactate dehydrogenase (LDH), pyruvate decarboxylase (PDC), pyruvate carboxylase (PC) and pyruvate dehydrogenase complex (PDHc). One unit (U) of enzyme activity for LDH and for PDC is defined as the amount of enzyme needed to oxidize (or reduce) 1 μmol of NADH per minute under optimal temperature and pH. On the other hand, one unit (U) of enzyme activity for PC and for PDH is defined as the amount of enzyme needed to generate 1 μmol of oxaloacetate per minute under optimal temperature and pH. Specific activity was expressed as the unit of enzyme activity per milligram of protein.
